# Variance explained by whole genome sequence variants in coding and regulatory genome annotations for six dairy traits

**DOI:** 10.1186/s12864-018-4617-x

**Published:** 2018-04-05

**Authors:** Lambros T. Koufariotis, Yi-Ping Phoebe Chen, Paul Stothard, Ben J. Hayes

**Affiliations:** 1 Queensland Alliance for Agriculture and Food Innovation, Centre for Animal Science, The University of Queensland, Building 80, 306 Carmody Road, Brisbane, St Lucia, QLD 4072 Australia; 2 Collage of Science, Health and Engineering, La Trobe University, Melbourne, VIC 3086 Australia; 3 Department of Economic Development, Jobs, Transport and Resources, AgriBio Building, 5 Ring Road, Bundoora, VIC 3086 Australia; 4 Dairy Bio, 5 Ring Road, Bundoora, VIC 3086 Australia; 5 Department of Agricultural, Food and Nutritional Science, University of Alberta, Edmonton, AB T6G 2C8 Canada

**Keywords:** Variance component analysis, Functional genomics, Enrichment or depletion analysis, Regulatory genome, Splice sites, DNA methylated regions

## Abstract

**Background:**

There are an exceedingly large number of sequence variants discovered through whole genome sequencing in most populations, including cattle. Deciphering which of these affect complex traits is a major challenge. In this study we hypothesize that variants in some functional classes, such as splice site regions, coding regions, DNA methylated regions and long noncoding RNA will explain more variance in complex traits than others. Two variance component approaches were used to test this hypothesis – the first determines if variants in a functional class capture a greater proportion of the variance, than expected by chance, the second uses the proportion of variance explained when variants in all annotations are fitted simultaneously.

**Results:**

Our data set consisted of 28.3 million imputed whole genome sequence variants in 16,581 dairy cattle with records for 6 complex trait phenotypes, including production and fertility. We found that sequence variants in splice site regions and synonymous classes captured the greatest proportion of the variance, explaining up to 50% of the variance across all traits. We also found sequence variants in target sites for DNA methylation (genomic regions that are found be highly methylated in bovine placentas), captured a significant proportion of the variance. Per sequence variant, splice site variants explain the highest proportion of variance in this study. The proportion of variance captured by the missense predicted deleterious (from SIFT) and missense tolerated classes was relatively small.

**Conclusion:**

The results demonstrate using functional annotations to filter whole genome sequence variants into more informative subsets could be useful for prioritization of the variants that are more likely to be associated with complex traits. In addition to variants found in splice sites and protein coding genes regulatory variants and those found in DNA methylated regions, explained considerable variation in milk production and fertility traits. In our analysis synonymous variants captured a significant proportion of the variance, which raises the possible explanation that synonymous mutations might have some effects, or more likely that these variants are miss-annotated, or alternatively the results reflect imperfect imputation of the actual causative variants.

**Electronic supplementary material:**

The online version of this article (10.1186/s12864-018-4617-x) contains supplementary material, which is available to authorized users.

## Background

The genetic component of complex trait variation, for many traits, is due to large numbers of mutations which individually explain a small portion of genetic variance [1–3]. GWAS using SNP genotype arrays with follow up studies have allowed for the detection of some of the mutations that underlie complex traits [1]. However, these types of analysis are limited in their power to detect causal mutations due to incomplete linkage disequilibrium and are limited to mutations that explain enough variance to exceed the high significance thresholds [1, 4, 5]. Further, the SNP from arrays used in GWAS are often biased towards common mutations [4], which leads to the issue that rarer genetic variants, that could have important effects on complex traits, may be undetected. Genomic prediction methods, where the effect of variants is estimated simultaneously to predict individuals genetic potential for phenotypes, can also be used to identify genetic variants associated with complex traits [6].

Whole genome sequencing (WGS) is providing solutions to some of the limitations of GWAS and genomic prediction with SNP genotyping arrays. In human and mouse genomes, the number of discovered genetic variants from WGS is well into the millions [7] and this technology is proving to be effective at finding a great number of previously unknown variants that are associated with traits and disease [8–10]. In livestock, such information is also proving useful for discovering both common and rare variants that have effects on complex traits, or cause disease [5, 11]. The 1000 Bull Genomes Project [12] has identified 28.3 million variants, including insertions & deletions (indels) and SNP. This has allowed for a greater resolution of sequence variants that can be imputed into large data sets for GWAS, or used in genomic prediction [13]. However, large number of variants at this magnitude are just too many to be used in genomic prediction due to computational limitations. Furthermore, in GWAS using such a large number of SNP could result in many variants with small effects to be missed, due to the high stringent significance threshold needed to avoid false positives with such a high degree of multiple testing [14]. In particular rarer variants, which explain only a small proportion of the variance may be undetected [15].

One strategy is to attempt to filter the large number of variants to a subset that is more likely to have effects [14, 16]. For example, underlying biological information could be used to identify variants in functional classes that have a priori associations with complex traits. Studies in humans and mouse have shown that annotating variants into functional classes can help to associate them with traits or diseases [1, 14, 16–18]. Functional classes involved with protein coding genes, such as missense mutations, are obvious candidates for prioritization since they are more likely to be enriched for trait associated variants [16–18]. Variants found in splice sites, should also be considered since studies have shown that they are good candidates for prioritization [5]. However, the majority of sequence variants are found outside protein coding genes, and it has been shown that regulatory classes such as, noncoding conserved regions, potentially methylated regulatory regions, miRNA, promoters and enhancers (in some cases identified by histone modifications and patterns of DNA methylation) can be enriched for variants significantly associated with complex traits [16, 17, 19–22].

Our hypothesis was to variants in some functional classes will explain more genetic variation, than expected by chance, and more variation than some other classes. Sequence variants from the 1000 Bull Genomes Project were annotated into 20 functional classes, including, but not limited too; target sites for DNA methylation regions (predicted from methylation patters in bovine placenta, which includes CpG island methylation and highly methylated regions [23], with the hypothesis that variants in these regions could disrupt the effectiveness of methylation), splice sites, synonymous, missense, long noncoding RNA (lncRNA), antisense RNA (asRNA) and untranslated regions (UTR). Out of these 20 annotation classes, 13 were used for further analysis (due to very small and large numbers of variants in some classes). Sequence variant genotypes were imputed into 16,581 dairy cattle with milk production and fertility phenotypes. To test our hypothesis, we performed two types of variance component analysis. The first analysis examined if variants in a functional class explain more variance than the variance explained by variants randomly chosen from a permutation test. In the second variance component analysis, genomic relationship matrices were constructed for each functional class and fitted simultaneously in the model, to partition the variance explained by each class.

## Results

### Annotation of full sequence variants

The 28.3 million sequence variants from Run4 of the 1000 bull genomes project were annotated into 20 functional classes (Table 1) based on their underlying biology derived from multiple data sources (Methods). Intron, intragenic and intergenic classes were not included for further analysis due to extremely large numbers of variants in these classes, therefore only 13 classes were used for further analysis.

**Table 1:** The annotated classes along with the number of variants in each class from the 28 million sequence variants

Class	Total Number of Variants	Percentage of WGS
3prime UTR	60,880	0.211%
5prime UTR	13,455	0.047%
Antisense RNA	14,198	0.049%
Exon coding sequence (CDS)	185,089	0.640%
DNA methylated regions in bovine placenta	204,702	0.708%
Downstream 5 k	731,297	2.531%
Exon	269,805	0.934%
Frameshift	93	0.000%
Intergenic	21,243,235	73.508%
Intragenic	6,961,936	24.091%
Intron	6,555,900	22.686%
Long noncoding RNA	147,025	0.509%
microRNA predicted target	79,205	0.274%
Missense deleterious	27,297	0.094%
Missense tolerated	71,908	0.249%
Splice site region	7988	0.028%
Stop codons	676	0.002%
Synonymous	105,598	0.365%
TFBS	8570	0.030%
Upstream 5 k	857,823	2.968%
Total	28,899,038	

The Percentage of WGS column represents the total proportion of annotated variants in each class as a percentage of the total WGS sequence variants. The majority of the annotations were obtained from Ensembl release 77 [44] except for the 3prime untranslated region (UTR), 5prime UTR, synonymous, missense deleterious and missense tolerated which came from the NGS-SNP pipeline [45]. MiRNA predicted target sites came from MicroCosm [46]. DNA methylated regions came from the study by Su J et al. [23]. Long noncoding RNA (lncRNA) and antisense RNA (asRNA) were obtained from the study by Koufariotis L et al. [49]. Transcription factor binding sites (TFBS) were from Bickhart D.M et al. [47]. Downstream 5 k and Upstream 5 k represent all variants that are found within 5 kilobases either upstream of a gene transcription start site (TSS) or downstream of a gene transcription termination site (TTS)

More than 70% of the sequence variants were located within intergenic regions (non-protein coding regions of the genome), and 25% were located within intragenic regions (protein coding genic regions). 23% of the variants were located within introns (constituting a total of 94% of the total intragenic variants). The number of annotated variants in each class (Table 1) were found to closely resemble the number of annotated variants from a study that deeply sequenced four unrelated Holstein dairy cattle [5].

Genotypes for the 28.3 million sequence variants were imputed into 16,581 dairy cattle, including cows and bulls, from the Holstein and Jersey breeds, with phenotypes for milk production and fertility traits (data described by Kemper et al. [24]. The phenotypes (trait deviations for cows and daughter trait deviations for bulls) were from the April 2013 genetic evaluations from the Australian Dairy Herd Improvement Scheme (ADHIS) and were for fat kg, milk kg, protein kg, fat percent, and protein percent and 15,667 phenotypic records were available for the trait fertility, Table 2.

**Table 2:** The phenotypes used in the analysis including the total number of records, or recorded phenotypes for each trait. From Kemper et al. [51]

Phenotype Name	Total number of Phenotypes	Number of Bull Phenotypes	Number of Cow Phenotypes	Number of Holstein records	Number of Jersey records
Fat Volume	16,581	4186	12,395	11,789	4792
Milk Volume	16,581	4186	12,395	11,789	4792
Protein Volume	16,581	4186	12,395	11,789	4792
Fat Percent	16,581	4186	12,395	11,789	4792
Protein Percent	16,581	4186	12,395	11,789	4792
Fertility (calving interval, days)	15,667	3999	11,668	11,040	4627

**Fig. 1:**
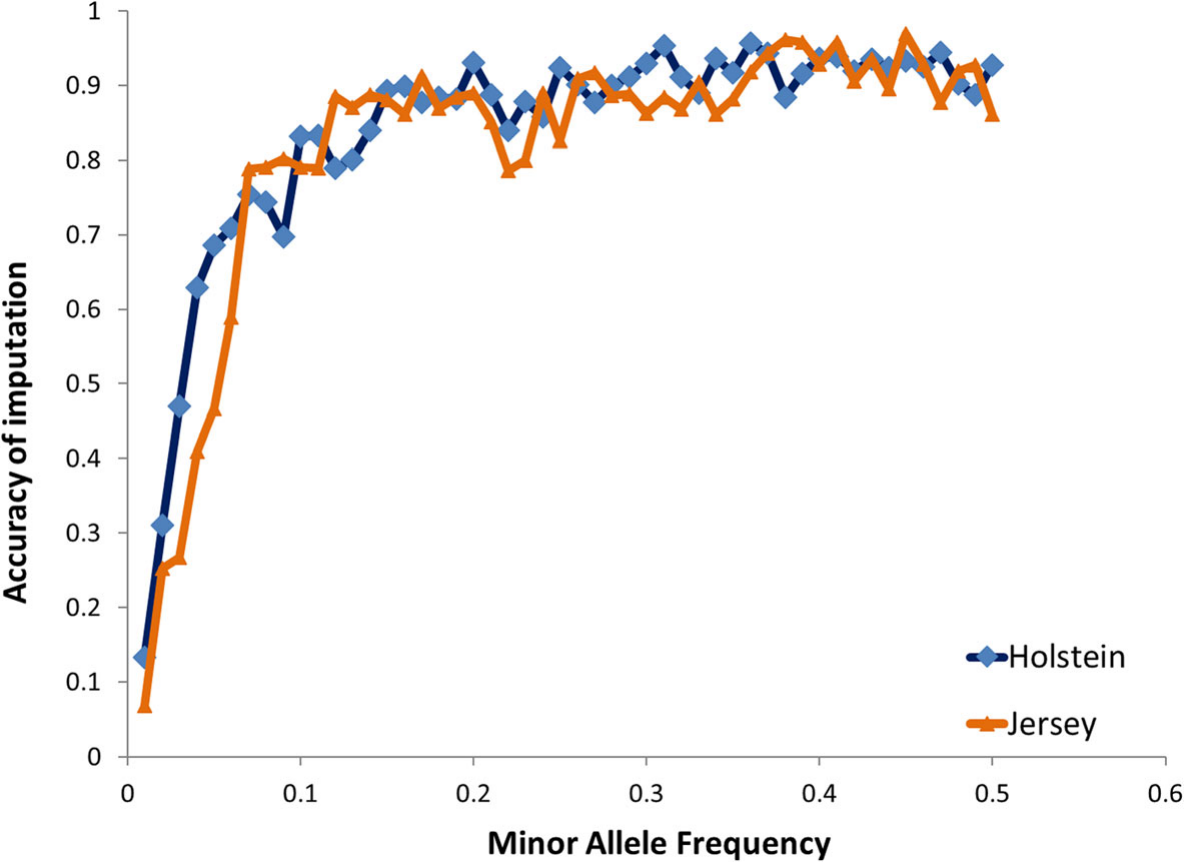
Accuracy of imputation. Accuracy of imputation to sequence variants in Holstein and Jersey cattle was assessed for sequence variants on chromosome 14. The accuracy of imputing genotypes was calculated from sequence genotypes of randomly selected 25 animals per breed from 1000 bull genomes Run4 were masked to those on the Illumina BovineHD BeadChip, then all the sequence variants were imputed using FImpute [25] with all other sequences (*N* = 1122) as a reference. Accuracy was the squared correlation between the imputed genotypes and true sequence variant genotypes

The accuracy of imputing sequence data was assessed for chromosome 14 (Fig. 1). Twenty-five animals of each breed (Holstein or Jersey) from 1000 bull genomes Run4 were masked to 800 K (Illumina BovineHD BeadChip), then all the sequence variants for these animals were imputed using FImpute [25] and all other sequences (*N* = 1122) as a reference. Accuracy was high when the minor allele frequency (MAF) > 0.1, however with low MAF the accuracy of imputation dropped rapidly.

### Allele frequency distributions

**Fig. 2:**
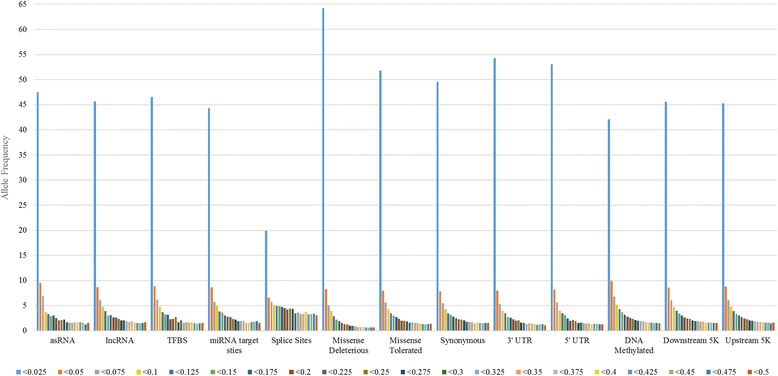
The MAF distribution for each annotation class. Each bar represents the number of variants found at that allele frequency. This is not cumulative however, as for each bar, the number of variants found is less than the allele frequency for that bar but greater than the allele frequency from the next bar. For example, the bar that represents < 0.5 represents variants in that class with a MAF less than 0.5 but greater than or equal to 0.475

The MAF distribution for sequence variants in each annotation class was calculated to determine differences in the allele frequency distributions between classes, Fig. 2 (Additional file 1). The majority of the variants in each class have very low allele frequencies, with 20–64% of the variants across all classes having an MAF of less than 0.025.

The missense deleterious, missense tolerated, 3′ and 5’ UTR classes have the largest proportion of variants with a MAF of less than 0.025, indicating that variants in these annotations may be under stronger selection than variants in other annotations. The missense deleterious class has the highest proportion of variants with a low MAF, in which more than 60% of the variants have an allele frequency of less than 0.025 (Fig. 2). This result has been observed in other studies [12].

Splice site regions, DNA methylated regions and miRNA predicted target site annotations have a much lower number of variants with a MAF of less than 0.025. In the DNA methylated regions (in bovine placenta) and miRNA target site classes, just over 35% of variants have a MAF between 0.1–0.5 while in the splice site region class more than 60% of the variants have a MAF between 0.1–0.5. The highest in this study.

There is a chance that variants that have very low allele frequencies could be sequence errors [12]. So, there is a trade-off in selecting a low enough MAF threshold to include most rare variants that can potentially be associated to traits, while simultaneously filtering out those more likely to be sequencing, or imputation errors. For this study we used a MAF threshold of 0.001 for filtering. We do acknowledge that this does not completely remove all errors and some rare variants could potentially still be sequence errors. Subsequent analysis in this study were also performed using a MAF threshold of 0.000000001 and 0.1. Results from these thresholds can be used to compare how the MAF filtering of including rarer variants (but higher chance of sequence and imputation errors) or including more common variants (lower chance of sequence and imputation errors) can impact the results of the variance component analysis. In general, the impact of the MAF threshold on the results was minimal.

### Variance component analysis 1: Genetic variance explained compared with a random permutation test

**Fig. 3:**
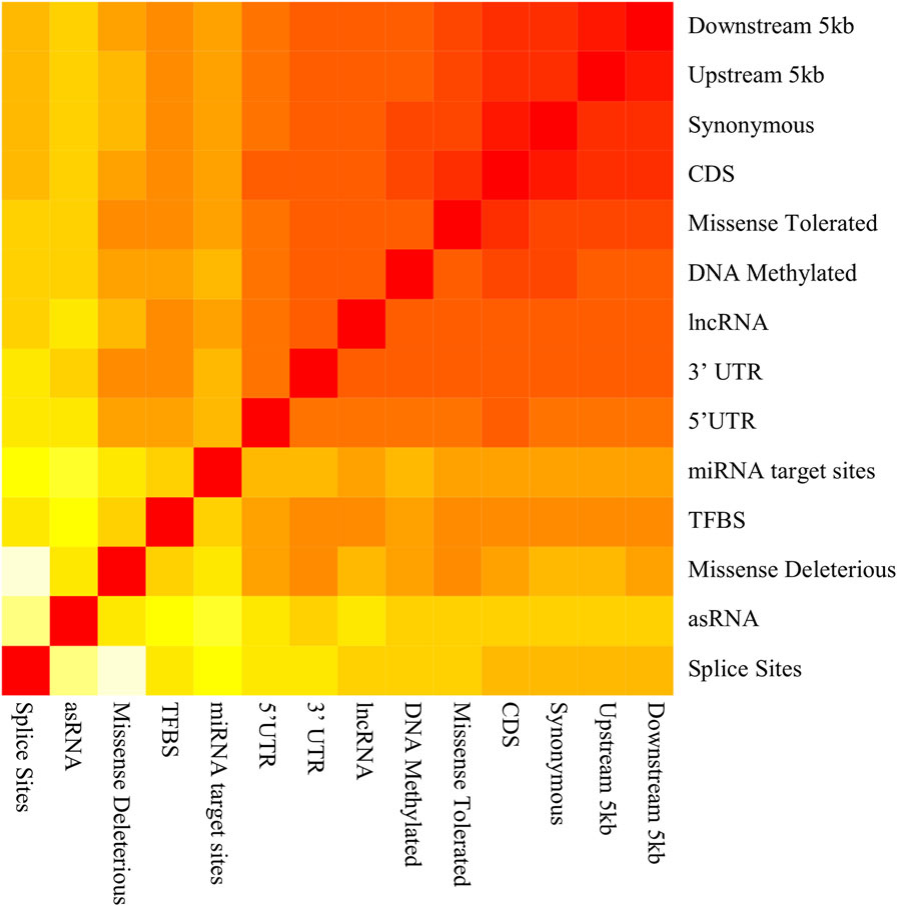
Heat map of the Euclidean Distance between the GRM for all classes. Heat map is ordered based on similarities between the annotation classes. GRM that are highly similar between each class are indicated with a red colour while the whiter the colour is the more dissimilar the GRM between each class is

Genomic relationship matrices (GRM) were constructed for each annotation class from the genotypes of all variants in the classes according to the method by Yang et al. [3]. To determine the similarities, or differences, between the GRM for each annotation class, we calculated the Euclidean distance between each pair of GRM, Fig. 3 (Additional file 2). The annotation classes are ordered based on their similarities (with highly similar GRM having a lower Euclidean distance). The GRM of the upstream and downstream classes are the most similar (Fig. 3), followed closely by the synonymous, CDS and the missense tolerated GRM. This is likely to reflect the high linkage disequilibrium (LD) between variants in these classes. The GRM for the missense deleterious and splice site classes had the least similarities with other genic classes, which can be due to the small number of variants found in these classes. The asRNA class shares little similarities with the genic classes nor with the upstream and downstream classes, a surprising result given that asRNA are known to overlap coding genes (but on the opposite strand). Possibly due to the small number of variants found in this class.

We next tested whether the variants in each annotation class explained more variance than expected by chance, give the number of variants in that class. This was done by obtaining the variance explained, using the GRM for each class, in a restricted maximum likelihood (REML) analysis using ASReml 4.1 [24] (Methods). To determine the variance explained by a random set of variants of the same number as found in that class, a variance component analysis was performed on the randomly selected variant subsets, using ASReml 4.1, to obtain the variance explained. This was performed a total of five times (the selection of random subsets and testing variance explained) to get a mean variance and standard error (S.E) for the randomly chosen variants, which was compared to the actual variance explained by the annotation classes. Variants were matched by allele frequency when random subsets were sampled. If the actual variance explained by the annotation class is significantly higher (given the standard error) than the variance explained by the random permutation set, we consider that class to be enriched for variants associated with, or affecting, the trait (Methods). This was performed for all 6 dairy traits.

**Fig. 4:**
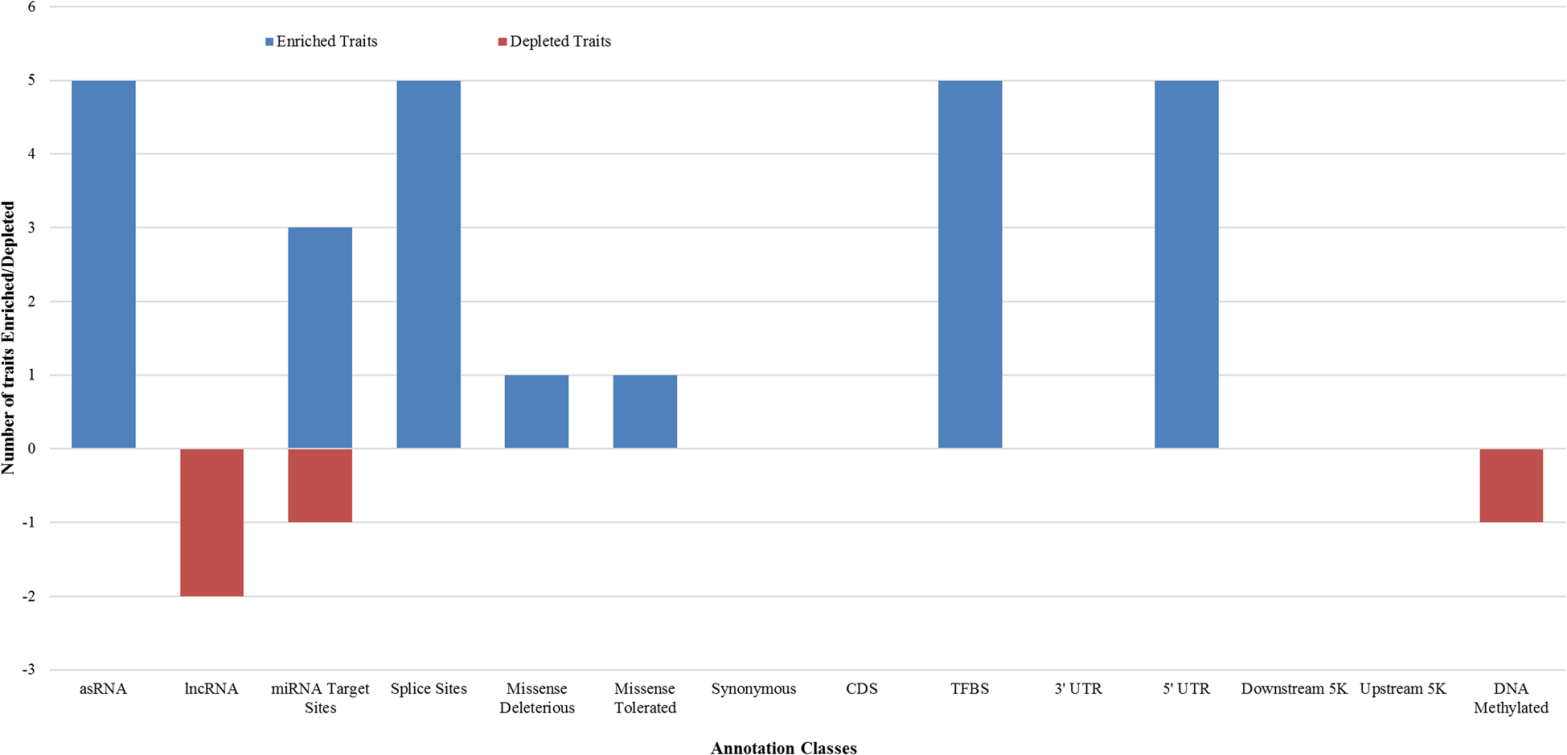
Enrichment/depletion analysis with permutation set. This graph shows the number of traits where variants in the annotation class explain significantly more, or significantly less of the variance than the same number of randomly selected variants (where the variance explained by chance is the average of five random subsets, and significance is determined by the standard error from these subsets). The total number of traits was six

Across most traits, variants in splice sites, asRNA, TFBS, and 5’ UTR classes all explained more variance than expected by chance (Fig. 4, Table 3). With the exception of fertility, all other traits had at least one class that explained significantly more variance than expected by chance (Fig. 4). The asRNA class consistently showed some of the greatest differences in the variance explained between the actual variance and the random permutation variance (Table 3, Additional file 3). Variants in the miRNA predicted target class for 3 traits (fat, milk and protein) captured more of the variance than expected by chance (Fig. 4, Table 3). However, this result was not consistent across traits, for the trait fat percent the miRNA class captured less variance than expected by chance. This was also observed (for fat%) in the lncRNA and DNA methylated regions in bovine placenta classes where less variance was captured. For the protein percent trait, variants in the lncRNA class captured less variance than expected by chance.

**Table 3:** The proportion of variance explained for each annotation class across all dairy traits

Class	Fat	Milk	Protein	Fat Percent	Protein Percent	Fertility
Antisense RNA	0.4 (0.15)*	0.48 (0.17)*	0.43 (0.15)*	0.6 (0.23)*	0.59 (0.2)*	0.02 (0)
Long noncoding RNA	0.28 (0)	0.34 (−0.01)	0.29 (0)	0.5 (−0.05)*	0.53 (−0.04)*	0.02 (0)
miRNA Target Sites	0.31 (0.04)*	0.37 (0.03)*	0.32 (0.04)*	0.5 (−0.02)*	0.53 (0)	0.02 (0)
Splice Sites	0.32 (0.03)*	0.39 (0.03)*	0.34 (0.03)*	0.49 (0.06)*	0.51 (0.07)*	0.01 (0)
Missense Deleterious	0.24 (−0.01)	0.31 (− 0.01)	0.26 (− 0.01)	0.41 (0.02)*	0.41 (− 0.01)	0.01 (0)
Missense Tolerated	0.27 (0)	0.34 (0)	0.28 (0)	0.5 (0.03)*	0.49 (0)	0.02 (0)
Synonymous	0.27 (0)	0.35 (0)	0.29 (0.01)	0.51 (0)	0.53 (0)	0.02 (0)
CDS	0.28 (0)	0.36 (0)	0.29 (0)	0.53 (0.01)	0.56 (0.01)	0.02 (0)
TFBS	0.37 (0.09)*	0.44 (0.09)*	0.39 (0.08)*	0.52 (0.11)*	0.52 (0.1)*	0.01 (0)
3’ UTR	0.27 (0.01)	0.34 (0.01)	0.28 (0.01)	0.48 (−0.01)	0.48 (−0.02)	0.02 (0)
5’ UTR	0.28 (0.03)*	0.34 (0.03)*	0.29 (0.02)*	0.45 (0.08)*	0.45 (0.06)*	0.01 (0)
Upstream 5 k	0.29 (0)	0.36 (0)	0.3 (0)	0.59 (0)	0.58 (−0.03)	0.02 (0)
Downstream 5 k	0.29 (0)	0.37 (0)	0.3 (0)	0.6 (0.01)	0.59 (−0.02)	0.02 (0)
DNA Methylated	0.28 (0)	0.34 (0.00)	0.29 (0)	0.54 (−0.03)*	0.57 (0)	0.02 (0)

In brackets is the difference in the variance explained (heritability, h^2^) between the actual variance explained in that class and the mean variance explained by five random permutation sets. An asterisk indicates a significant difference between the variance explained by that class and the random permuted test. DNA methylated represents methylated regions in bovine placenta [23]. 3 prime and 5 prime untranslated regions (UTR) are represented as 3’ UTR and 5’ UTR, respectively. CDS represents the coding sequence of an exon. Transcription factor binding sites (TFBS) were from Bickhart D.M et al. [47]. Downstream 5 k and Upstream 5 k represent all variants that are found within 5 kilobases either upstream of a gene transcription start site (TSS) or downstream of a gene transcription termination site (TTS)

For the downstream and upstream classes there were no traits for which the variance explained was significantly greater than expected by chance (Table 3). This result is surprising, given that in previous studies many traits in these classes were found to be significantly enriched for trait associated variants (TAVs) [16]. This result is possibly due to the fact that there are a very large number of variants in these classes, (731,297 downstream class and 857,823 upstream class), and with such a large number of variants, most of the genetic variance for the traits will be captured with the random subset of the same number of variants. That is, the LD with causative mutations will be reasonably high even in the randomly selected variants, thus making it difficult to determine any additional variance explained by the functional class. For classes that consist of a smaller number of variants, such as the TFBS and the asRNA, the LD with all other variants in the genome is lower, as they are less likely to be evenly spread across the genome. This analysis is best applied to classes with a smaller number of variants as there is more power in finding the difference in the amount of variance captured by the annotation class vs the randomly chosen variants from the permutation test.

Variants annotated in the CDS and synonymous classes did not explain more variance than expected by chance across most traits, however, the CDS class does slightly explain more variance in fat percent and protein percent (Table 3), while the synonymous class explains slightly more variance in protein (Table 3). This is another surprising results given that in a previous study these classes were significantly enriched for TAV [16]. We postulate the reason for this is probably due to the considerable number of variants found in these classes that explain most of the variance and are likely to be in higher LD with causative mutations. The full table that includes the actual variance explained (heritability) for each functional class, along with the variance explained by the random permutation test and the heritability difference is provided in Additional file 3.

### Variance component analysis 2: Capturing the proportion of variance explained when variants in annotation classes are fitted simultaneously

**Fig. 5:**
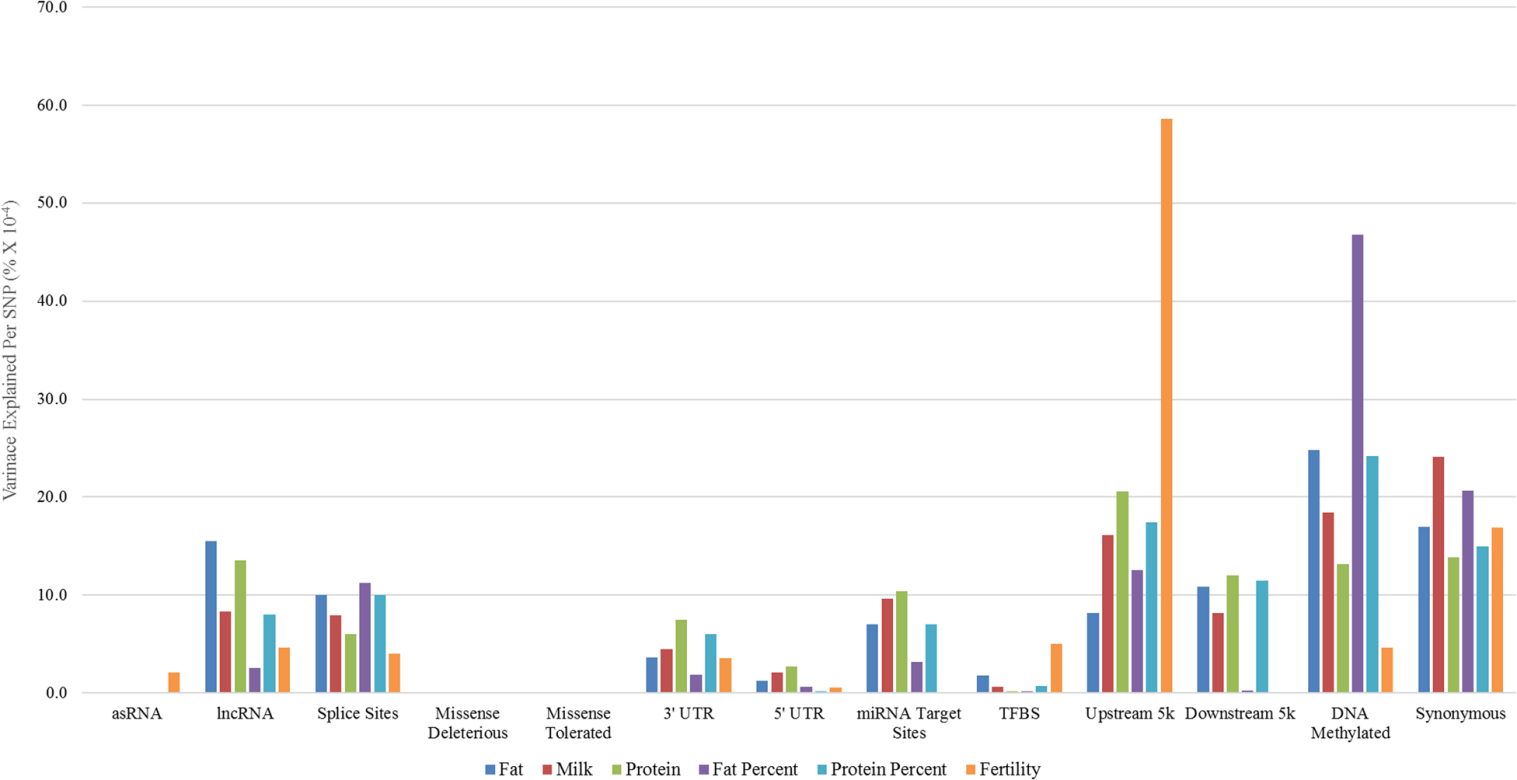
The proportion of variance captured by each annotation class. The proportion of variance captured by each annotation class is calculated by fitting the GRM simultaneously in the REML model to determine the variance explained by each class when in the presence of another class

One limitation of the approach taken above, is that due to the very large number of variants found in some annotation classes, and the extensive LD in the cattle population, power to detect additional variance explained over and above that expected by chance is limited. To overcome this, a second variance component analysis was performed fitting the GRM for each functional class simultaneously in the model to capture the variance component from each class when in the presence of all other classes. We also determine the variance explained per sequence variant (Methods) to measure how much variance each variant capture in a class. To perform this analysis, GCTA [26] was used to fit all GRM simultaneously in the REML mode. The proportion of the variance captured by each functional class of SNP is shown in Fig. 5.

The synonymous, DNA methylated regions and upstream classes capture the highest proportion of variance across most traits, Fig. 5. Variants in the upstream class captured close to 60% of the variance in fertility (Fig. 5). For the DNA methylated class, the total proportion of the variance captured is close to 50% in fat percent, while for other traits, 14–24% of the variance was captured by variants in this class. As for the other regulatory classes; the lncRNA, miRNA target sites and downstream classes capture a modest proportion of the variance across most traits, except for fat percent and fertility in the downstream class. With the exception of fertility, where only a very small amount of variance was captured, the proportion of variance explained by the asRNA class is negligible, a surprising result given that in the previous variance component analysis, this class explained significantly more variation than expected by chance (Fig. 4).

Out of the intragenic classes, the synonymous class captures the highest proportion of the variance for all traits, followed by the splice site class; in which the average proportion of the variance captured across traits was 8%. For variants in the missense deleterious and missense tolerated classes, the proportion of variance captured was, unexpectedly, almost nil. In the previous variance component analysis, comparable results were observed, except for fat percent (Fig. 5). These results may reflect low MAF for variants in these classes, or imputation and sequence errors which are more likely for low MAF variants. The 3’ UTR and 5’ UTR classes capture a modest but small proportion of the variance.

**Fig. 6:**
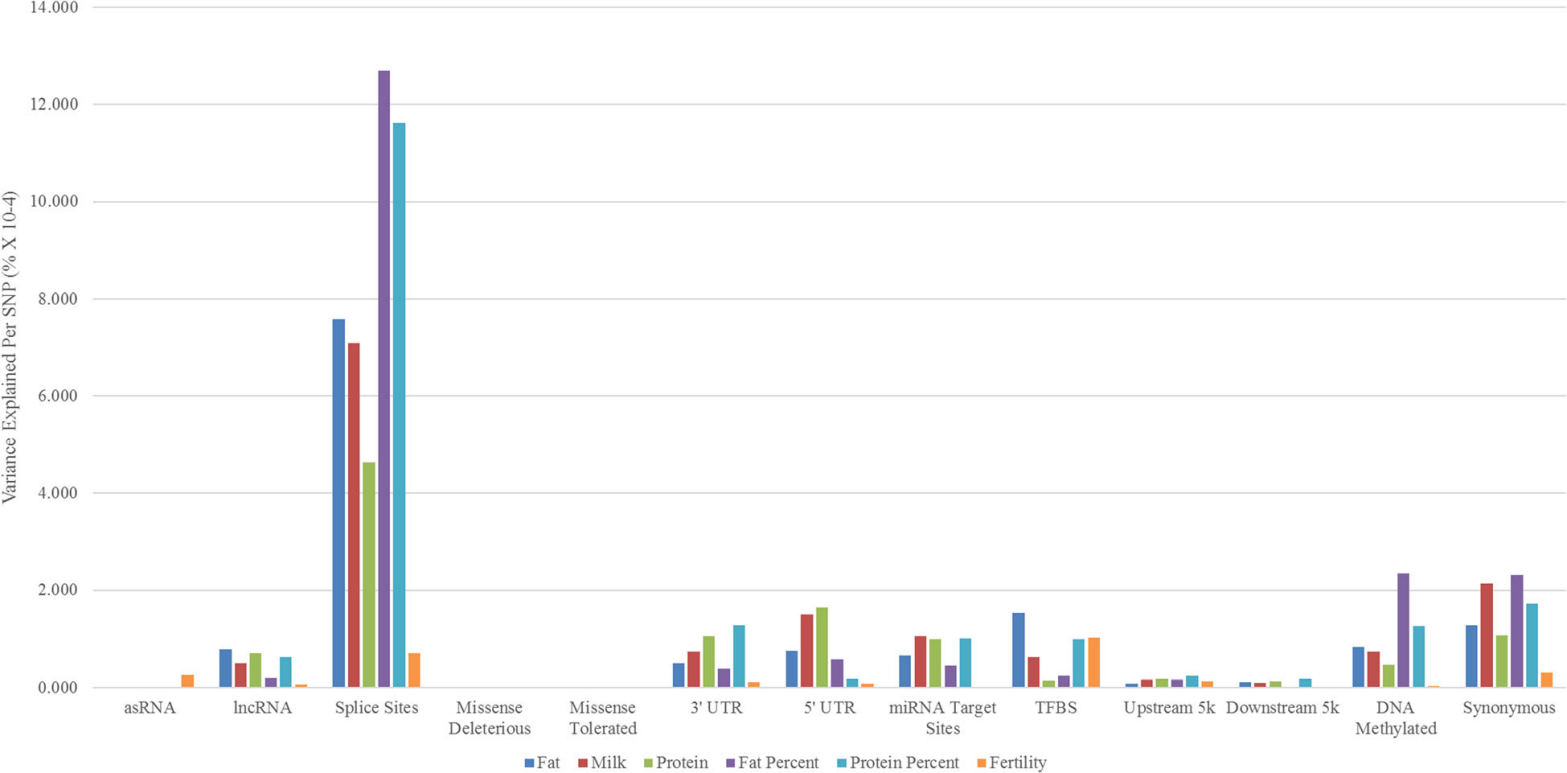
Proportion of variance explained per sequence variant for each annotation class. Each bar represents a single trait. On the x-axis are the annotation classes and the y-axis is the proportion of the variance captured per sequence variant (as a %). This was calculated by dividing the proportion of variance explained by each class by the number of variants in that class

As the number of variants in each class varies greatly, we investigate how much variance is explained (on average) by each individual variant for all classes, Fig. 6. This was calculated by dividing the proportion of variance explained by that class with the total number of variants in that class. In this analysis, we find that the splice site class had the largest variance explained per variant for all traits except fertility, providing evidence that this class contains variants that can contribute to trait variation and should be prioritized in further studies. Out of the intragenic classes; the synonymous, 5′ and 3’ UTR classes explain a modest amount of variance, per sequence variant, for all traits except fertility. Consistent with the results from the previous analysis (Fig. 5) and surprisingly, the missense deleterious and missense tolerated classes again explain almost no variance.

Out of the regulatory classes, the DNA methylated class explained most of the variance per sequence variant in fat percent and protein percent. While for variants in the miRNA target sites class, we observe a modest proportion of the variance explained per variant, particularly for fat, milk and protein. The variance explained per sequence variant in the lncRNA and TFBS was also relatively modest, particularly for the traits fat, protein percent and fertility, which explain slightly more variance than that in lncRNA. The upstream and downstream classes, on the other hand, capture very little of the variance per sequence variant (Fig. 6) which does deviate from the results obtained in the previous analysis (Fig. 5). We assume this is due to the very large number of variants found in these classes, where only a small number are likely to be causative mutations - because the variance explained is spread equally among all the variants in the classes, it also includes the nonfunctional variants that have little to no effects which are likely to be abundant in the upstream and downstream classes. This leads to the true variance explained, per sequence variant, by these to be quite low.

As with the previous analysis, a MAF threshold of 0.001 was used to filter out sequencing and imputation errors. The above analyses were also performed using an MAF threshold of 0.000000001 and 0.01, and those results can be found in Additional files 4 & 5. Additional file 4 contains the proportion of variance captured by each class when using these MAF thresholds, while Additional file 5 contains the variance explained, per sequence variant, by each class when using these MAF thresholds. There was minor difference in results with the different thresholds.

### Variance component analysis 2: Comparison of results with cow and bull data sets

Cow and bull phenotypic records were available for the traits milk volume, fat volume, protein volume, fat percent, protein percent and fertility. The bull dataset includes records from thousands of cows to obtain the daughter trait deviations, the majority of which are not present in the cow dataset. Therefore, the cow and bull datasets are very close to independent, and we can use them to cross-validate the results (given we expect to see few sex specific differences). As with the previous analysis, the GRM for all functional classes was fitted simultaneously in the model, only this time, the REML was calculated using phenotypic records strictly from either cow or from bulls. Both the proportion of variance captured and the variance explained per sequence variant for each class was recorded for the cow and bull phenotypic records.

**Fig. 7:**
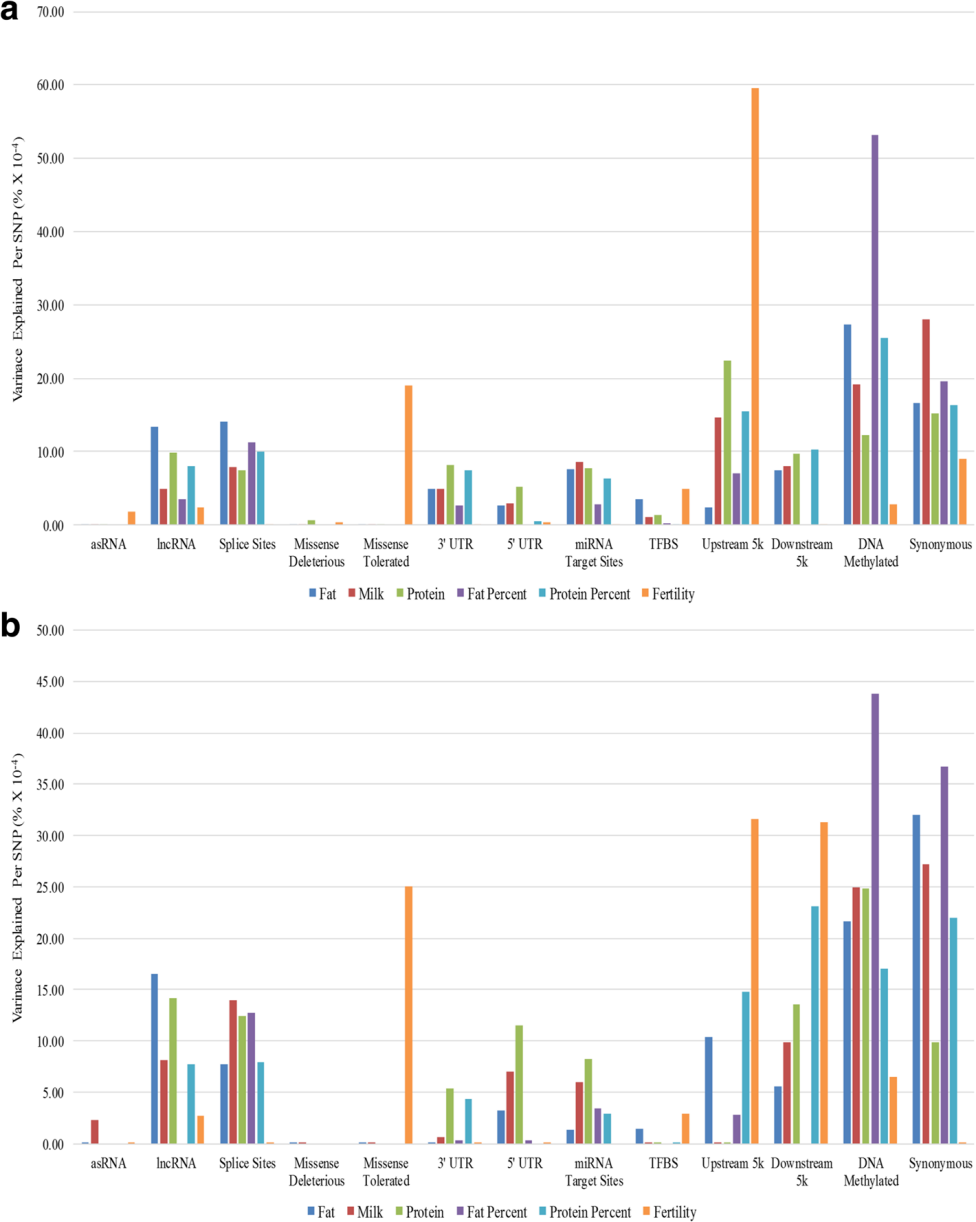
The proportion of variance captured by each class for cow and bull records. The x axis represents the functional classes, the y axis representing the proportion of variance captured by each class (as a percentage of the total variance explained in that trait). **a** Only cow phenotypic records (**b**) only bull phenotypic records

Overall both sexes follow very similar trends in the proportion of variance captured by each class (Fig. 7a and b), and the results were similar to the results in the previous analysis with all animals (Fig. 5). Notable differences can be seen in the upstream class for fertility, where bull records capture just over 30% of the variance while cow records capture close to 60% of the variance, which resembles the proportion of variance captured in the previous analysis, Fig. 5. We additionally observe a very similar pattern for fertility in the downstream class, where the proportion of variance captured by the cow records is minimal, (similar to what we observe in Fig. 5), however bull records capture close to 32% of the variance.

## Discussion

Whole genome sequence variants were annotated into 13 functional classes, then genotypes for these variants were imputed into a large population of dairy cattle with milk and fertility phenotypes, to test the hypothesis that variants in some annotation classes will explain more variance than others for these traits. We find for most traits, the asRNA, splice site regions, 5’ UTR, miRNA target sites and TFBS classes explained more variation than expected by chance, given the number of variants in these classes. The second variance component approach fitted the GRM constructed from variants in each class simultaneously. With this approach it was observed that variants in DNA methylation target regions (at least in bovine placenta), synonymous and upstream classes captured a considerable proportion of the variance. On a per sequence variant basis, variants in the splice site regions had the highest proportion of variance explained per sequence variant for most traits. Finally, we assessed bull and cow phenotypes in separate analyses to investigate any sex differences; finding that there was little evidence for this.

Many authors propose that variants found in coding regions (intragenic variants) have the greatest potential to be functionally important and more likely to contribute to trait variation [27]. Out of the variants annotated in intragenic regions, the splice site and synonymous classes are some of the most significant in our study, capturing a large proportion of the variance out of the total variance explained (Fig. 5) and also per sequence variant (Fig. 6). Variants found in splice site regions are of particular interest, as significantly more variance was explained by variants in this class than expected by chance (Fig. 4). Other studies have found that SNP in splice site regions are significantly associated with traits [28], supporting our findings. The study by Li et al. [29] has provided additional evidence of the importance that splicing patterns have on trait variation, finding that splicing quantitative trait loci (QTL) have major contributions to complex traits in humans, in fact, these contributions are stated to be just as significant as variants that affect gene expression [29]. We provide evidence that variants in splice site regions, are not only common, but have the potential to be of great importance for prioritization in further studies.

The most unexpected results in this study is the little to no variance captured by the missense deleterious and tolerated classes (Fig. 5). Only for a single trait was tis annotation class found to capture more variance than expected by chance (Fig. 4), while fertility showed a modest proportion of the variance captured in the validation analysis using bull and cow records (Fig. 7a and b). Missense variants are expected to have effects on traits since they alter the polypeptide sequence of a protein, and their significant association with traits have been shown by similar functional studies [16–18]. One possible explanation for our results is that rare missense mutations are more likely to be deleterious [30] and over time their frequencies are kept at very low levels in the population due to purifying selection [31]. Another possibility is due to how the GRM is calculated from a subset of variants that have very low frequencies. The missense classes, particularly the missense deleterious class, have some of the highest proportion of variants with low MAF and calculating the GRM with such a large number of variants with low MAF reduces the accuracy in estimating the variance component [12]. This in turn affects how much variance is truly captured.

Another explanation is due to the potentially larger number of sequence and imputation errors present in the missense classes, given the low MAF for this class of variant. As shown in Fig. 1, the accuracy of imputation is low for rare variants. Inaccurately imputed variants, might have little to no association with traits and thus can potentially reduce the significance of the class, even if the class really does include a reasonable proportion of mutations affecting the traits.

The synonymous class, surprisingly, captured a much greater proportion of the variance than the missense deleterious class did for all traits. There are a number of possible explanations for this observation. Some studies have found that synonymous mutations are enriched for trait associated variants [16, 17], with research suggesting that these mutations are not silent, rather they are associated with altering protein expression, conformation, function and are even believed to have codon usage bias [32]. Further, synonymous mutations are believed to be under purifying selection, particularly if they overlap regions of the genome that are involved in motif binding [33]. In fact, the study by Chen et al. [34], finds that synonymous SNP are just as likely as non-synonymous SNP to be associated with disease traits, and should be included in further functional studies. It is also possible (and actually more likely) that most of these variants are in high LD with nearby causative mutations that could be either unannotated, or share a very similar allele frequency [35] which are contributing to how much variance this class explains. A final explanation is that, due to imperfect imputation of actual causative mutations (which may be missense mutations), some of the synonymous variants are in higher LD with the causative mutations than the imputed causative mutations themselves.

Human studies find that phenotypic variation for complex traits occurs in regulatory regions and highly conserved regions [21, 22]. Our results support this notion that variants in regulatory classes can be just as significant as variants within protein coding genes. Variants in regulatory classes such as target sites for DNA methylation, upstream, downstream, lncRNA and miRNA target sites all explained moderate to large proportions of the variance for most traits (Fig. 5). Notably, variants found in DNA methylated regions (at least in bovine placentas) and upstream regions captured some of the highest proportions of the trait variance. Other research has also found that these classes are significantly associated with traits [16, 18, 36]. This is counterintuitive, as DNA methylation is associated with turning off, or limiting, gene expression. So why would a variant in DNA methylation target site affect a complex trait? One possibility is that some of these variants disrupt the effectiveness of methylation, resulting in increased or altered gene expression, ultimately affecting complex trait phenotypes [37, 38].

The lncRNA and asRNA classes are known to have important regulatory functions in the cell [39] and mutations can potentially lead to altering their primary or secondary structure, or lead to abnormal expression which can affect how genes are regulated [40, 41]. Our results demonstrate that although some traits explain less variation by the lncRNA class, compared to the other classes, given the number of variants in this class a modest proportion of the variance is explained for several traits (Fig. 4). For the asRNA class, most traits captured significantly more variation than expected by chance with the Variance component analysis 1 (Fig. 4), however the total proportion of variance explained (Variance Component Analysis 2) is minimal (Fig. 5). The likely reason why this occurs is due to the small number of variants in this class, which can impact the total variance explained. These results show that lncRNA can potentially be associated with dairy traits through a regulatory role and further studies into the function of bovine lncRNA are needed.

The study by Das et al. [5] is one of the few studies that functionally annotate bovine sequence variants from WGS to determine if functional classes of SNP are associated with traits. In that study, a total of 10,796,794 SNP were discovered, of which 2145 were found to be loss of function variants. Further, more than 60% of loss of function variants have a MAF of greater than 0.05 when using the 1000 bulls genome data set [5]. The proportion of annotated variants that were found to be intergenic (68.0%), synonymous (0.4%), intron (24.6%) and 3’ UTR (0.2%) were highly correlated with our results as seen in Table 1. Using a gene ontology enrichment analysis approach Das et al. [5] found that loss of function variants are overrepresented in genes involved in olfactory receptor activity and G-protein coupled receptors among many others [5]. This provides some evidence that loss of function variants, including splice site variants (either through insertion, deletions or SNP at a splicing site can alter the processing frame and lead to a loss of function of the mRNA and final polypeptide transcript), are significantly associated with traits. In another study by Finally this study is an extension to the study by Koufariotis et al. [16] in which annotated SNP from the 777 K Bovine HD array were used to determine if functional classes of SNP are associated with dairy and beef traits. In that study it was concluded that the synonymous and missense mutations explain the largest proportion of variance, per SNP, and many traits were significantly enriched for TAVs. Further, significant enrichment for TAVs was observed in the 5 Kb upstream and 5 Kb downstream classes [16].

A limitation in the variance component analysis 2, when all GRM across all classes were fitted simultaneously, we observed that different complex traits are affected by different annotation classes. One example of this is the trait fertility, which seems to be significantly affected by variants in the upstream class (Fig. 3). These results are peculiar as we would expect to find similar enrichment patters across all functional annotation classes. A possibility for this could be that for some traits, such as fat percent, are impacted by a few major mutations that have large effects, and these major mutations will be found in a certain annotation class. This will skew the results to show that certain annotation classes have greater affects. Another possible explanation is that some complex traits might have low heritability, such as fertility. Lastly, it could also reflect a real difference between traits, and that variants in certain genomic regions do affect specific traits more so than other traits, however, more data would be needed to prove that this is the case.

One important limitation that impacts this study has to do with the state of annotation of the bovine genome. Compared to human and mouse genomes, the current annotation state of the bovine genome is relatively poorly characterized [42]. This leads to the possibility that some variants can be incorrectly annotated, particularly for variants located in close proximity to each other, or near “borders” such as the intron/exon borders. Further, if the coding frame is not reliably annotated, some variants might be miss-annotated, for example, a missense SNP might be incorrectly annotated as a synonymous SNP. This limitation, can potentially affect the total variance explained by some classes in this study by “reducing” the variance explained, especially if there is a larger number of miss-annotated variants in the class. Additionally, it can also lead to an “increase” in the variance explained if an annotation class includes miss-annotated functional SNP. The FAANG (functional annotation of animal genomes) project is aiming to provide an ENCODE style approach to produce a comprehensive data resource of functional annotations in livestock genomes, including cattle [42].

## Conclusion

This study has shown that by using variance component analyses, sequence variants annotated in certain classes explain more of the variance than expected by chance, given the number of variants in the class. In addition to this, variants annotated in some of these classes explain substantially more trait variance on a per sequence variant basis (when variants from all classes are fitted simultaneously). Many regulatory classes, particularly sites that have been observed to be methylated in some cases, lncRNA, miRNA target sites and TFBS captured modest to large proportions of the variance. The synonymous and splice site variants captured some of the highest proportions of the variance out of the protein coding classes. Further, the splice site class captured the greatest proportion of variance, per SNP, for most traits. We propose that splice site variants, and RNA splicing, should be of greater focus in future work to understand the associations these variants have in complex dairy traits. A limitation in the current study was the accuracy of imputation, particularly of variants with low MAF, and it is important to recognize this may have had an impact on our results, particularly for those annotation classes with many low MAF variants, such as the missense class.

## Methods

### Whole genome sequence variants

Sequence genotypes from real and imputed 800 K SNP Chip array genotypes (2,450,800 K genotypes, the rest of the 16,581 were 50 K genotypes imputed up to 800 K with an accuracy of 0.98) were imputed using Fimpute software [43] into full sequence datasets. The reference genome sequences used for imputation were from Run4.0 of the 1000 Bull Genomes Project [12] which included 1148 *Bos Taurus* sequences from a range of dairy and beef breeds (including 311 Holstein and 61 Jersey bulls). A total of 28.3 million sequence variants were available for this study.

### Annotation of variants using Ensembl databases

Sequence variants were annotated into the following classes; intergenic, intragenic, exon, intron, CDS, 5 kb upstream of a transcription start site, 5 kb downstream of a gene, frame-shift variants, splice site region variants and stop codons classes by querying the Ensembl variant database version 77 [44]. Splice site variants includes those that are annotated by Ensembl as either splice acceptor variant (a variant found near the 3′ end in an intron) a splice donor variant (a variant found near the 5′ end of an intron) and all other variants annotated as splice region variants in Ensembl. Not all the variants found in splice regions will actually alter splicing.

### Annotation of variants with NGS-SNP

The classes missense deleterious, missense tolerated, synonymous, 3’ UTR, 5’ UTR were annotated in a previous study using the NGS-SNP tool [45]. We queried the NGS-SNP annotated sequence variant database for annotation of the variants in the above classes.

### Annotation of microRNA predicted target sites and transcription factor binding sites

MicroRNA predicted target sites were obtained from the MicroCosm target site database [46]. TFBS were obtained from the study by Bickhart et al. [47], the TFBS genome positions were converted from Bau4.1 assembly to UMD3.1 assembly using the UCSC LiftOver tool and queried the TFBS positions for sequence variants.

### Annotation of DNA methylated regions/sites

The DNA methylated regions were obtained from the paper by Su et al. [23] that predicted a bovine DNA methylation map using a combination of high-throughput sequencing and methylated DNA immunoprecipitation form bovine placental tissue. In that paper, they found evidence of highly methylated regions which covered 5.86% - 5.89% of the genome, including methylated genes (defined when the overlap between a gene and a highly methylated region is greater than 50%) and methylated CpG islands (defined when a CpG island overlaps with a highly methylated region). We took the genomic locations of the highly methylated regions and the methylated CpG islands from that study for both control and somatic cell nuclear transfer clone placentas. From these, we determined the total number of sequence variants located within the highly methylated regions and methylated CpG islands and categorized them in a single annotation class; DNA methylated regions.

Patterns of DNA methylation have been reported to remain static between tissues and throughout the life of a cell [48], however it has been suggested that DNA methylation patterns in the placental tissues can be highly variable when compared to other tissues [25]. The degree of differentiation in DNA methylation between individuals and tissues is still an area that requires much research. In this paper, we acknowledge that (some of) the DNA methylated regions obtained by Su et al. [23] will be specific to placental tissues, but this is the most comprehensive data set of its type to date.

### Annotation of long noncoding RNA and antisense RNA

Long noncoding RNA were obtained from the study by Koufariotis et al. [49] and from the domestic-animal lncRNA database (ALDB) [50]. Both the lncRNA from Koufariotis et al. [49] and ALDB were queried to determine if sequence variants are found within the lncRNA start and end on the genome.

### Variance component analysis 1: Determining genetic variance with random permutation test

The variance component analysis was performed to determine if SNP in a functional class explain more variance than the variance explained by the same number of randomly selected variants from a random permutation test. The genome relationship was calculated for each functional class of SNP according to the Yang et al. method [3].

To measure the similarities between each GRM for all functional class of SNP (to determine if variants are common between two functional classes), the Euclidean distance was calculated using the following formula:

$$ \mathrm{Euc}.\mathrm{Dist}=\sqrt{\sum \limits_{\mathrm{i}=1}{\left({\mathrm{m}}_{\mathrm{i}}-{\mathrm{p}}_{\mathrm{i}}\right)}^2} $$

Where *m* and *p* is the corresponding GRM for each class.

To calculate the proportion of variance explained by each functional class of SNP, a REML analysis was performed by fitting the following model to the data

$$ y= xb+ zg+e $$

Where *y* denotes a vector of the dairy phenotypic records obtained from the following study [51], and these phenotypes were weighted, in the case of cows by the number of repeated records, and in the case of bulls by the number of daughters (as described in [24], *b* is a vector of fixed effects that includes the breed and sex, *x* is the design matrix that allocates the records to the fixed effects, *z* is design matrix that allocates records to breeding values and *g* denotes a vector of random breeding values obtain from the following formula:

$$ g\sim N\left(0,G{\sigma}_g^2\right) $$

Where g is the GRM for the functional classes of SNP, and ${\sigma}_g^2$ is the genetic variance from each functional class. ASReml version 4.1 [24] was used to estimate the proportion of phenotypic variance (heritability, h^2^) from the above models.

The random permutation test involves calculating the variance explained from a randomly chosen set of *n* SNP, where *n* corresponds to the number of variants found in the functional class while selecting for variants that had similar allele frequencies as the functional class they represent. For each class, the same number of variants was randomly selected from the sequence variant dataset and the GRM was calculated from the random set. ASReml 4.1 [24] was used to fit the same formula as above to calculate the phenotypic variance explained (heritability) by the randomly selected variants. This random sampling was run a total of 5 times, with each iteration selecting for different random variants, to get a standard error and the average of these 5 runs was calculated to obtain the random set variance explained.

To calculate the significance of the enrichment or depletion of a class (Table 4), the percent difference of the variance explained by the random permutation test and the actual variance explained by the class was calculated using the equation below:

$$ percent\ difference=\left(\frac{h^2-{rh}^2}{rh^2}\right)\times 100 $$

**Table 4:** The variance components for each class across all traits when fitting the GRM simultaneously in the model.

Class	Fat (kg)	Milk (kg)	Protein (kg)	Fat Percent*	Protein Percent*	Fertility (calving interval, days)
AntisenseRNA	0 (0%)	0.69 (0%)	0 (0%)	0 (0%)	0 (0%)	1.63 (2.1%)
Long noncoding RNA	24.51 (15.5%)	12,041.50 (8.3%)	14.09 (13.6%)	0.14 (2.6%)	0.1 (8%)	3.67 (4.6%)
Splice Sites	15.85 (10%)	11,474.41 (8.0%)	6.21 (6%)	0.61 (11.3%)	0.13 (10%)	3.16 (4%)
Missense Deleterious	0 (0%)	0.69 (0%)	0 (0%)	0 (0%)	0 (0%)	0 (0%)
Missense Tolerated	0 (0%)	0.69 (0%)	0 (0%)	0 (0%)	0 (0%)	0 (0%)
3’ UTR	5.74 (3.6%)	6492.31 (4.5%)	7.78 (7.5%)	0.10 (1.9%)	0.08 (6%)	2.83 (3.6%)
5’ UTR	1.99 (1.3%)	3078.63 (2.1%)	2.80 (2.7%)	0.04 (0.7%)	0.002 (0.2%)	0.45 (0.6%)
miRNA Target Sites	11.07 (7.0%)	13,887.10 (9.6%)	10.78 (10.4%)	0.17 (3.2%)	0.1 (7%)	0 (0%)
TFBS	2.78 (1.8%)	874.09 (0.6%)	0.16 (0.2%)	0.01 (0.2%)	0.01 (0.7%)	3.97 (5%)
Upstream 5 k	12.85 (8.1%)	23,201.91 (16.1%)	21.37 (20.6%)	0.67 (12.6%)	0.22 (17.4%)	46.31 (58.6%)
Downstream 5 k	17.23 (10.9%)	11,835.03 (8.2%)	12.47 (12%)	0.01 (0.2%)	0.15 (11.5%)	0 (0%)
DNA Methylated	39.11 (24.8%)	26,529.43 (18.4%)	13.68 (13.2%)	2.51 (46.7%)	0.30 (24.2%)	3.65 (4.6%)
Synonymous	26.76 (16.9%)	34,816.95 (24.1%)	14.37 (13.9%)	1.11 (20.7%)	0.19 (15%)	13.35 (16.9%)
Error (Ve)	129.10	78,206.27	80.22	5.36	1.25	534.81

In the brackets is the proportion of the total variance captured by variants in the class for the trait. The traits with an asterisk had their variance component multiplied by 100 since the values where too small to display in 2 decimal places. DNA methylated represents methylated regions in bovine placenta [23]. 3 prime and 5 prime untranslated regions (UTR) are represented as 3’ UTR and 5’ UTR, respectively. CDS represents the coding sequence of an exon. Transcription factor binding sites (TFBS) were from Bickhart D.M et al. [47]. Downstream 5 k and Upstream 5 k represent all variants that are found within 5 kilobases either upstream of a gene transcription start site (TSS) or downstream of a gene transcription termination site (TTS)

Where *h*^*2*^ denotes the actual variance explained (heritability) for a functional class of SNP and the *rh*^*2*^ represents the variance explained from the random permutation test for each functional class, which is the average heritability obtained by the 5 iterations of the random permutation test.

### Variance component analysis 2: Total proportion of variance explained when variants in annotation classes are fitted simultaneously

In the previous analysis, classes that have very large numbers of variants will capture most of the heritability for each trait, regardless if the variance explained is from the actual functional class being tested, or from the random permutation test set. This makes it particularly difficult to determine the true additional variance explained. To get around this, the total proportion of genetic variance a functional class explains was determined by fitting the GRM from all functional classes simultaneously in the model. The tool Plink [52] was used to prepare and modify genotype files to convert them into binary format that can be used by the tool GCTA [26]. The GRM for each class was calculated using GCTA and a REML analysis was performed by fitting the GRM for all classes simultaneously in the model (with GCTA) to obtain the phenotypic variance for each class. The ratio of genetic variance to phenotypic variance was recorded for each trait along with the standard error. To calculate the total proportion of variance captured by each trait for all classes the following formula was used:

$$ total\ proproprion\ of\ variance=\left(\frac{h^2}{\sum_{i+1}\left({h^2}_i\right)}\right)\times 100 $$

Where *h*^*2*^ represents the variance captured by each functional class, divided by the total sum of all the variance captured for each trait and multiplied by 100.

Further, to calculate the variances explained on a per sequence variant basis the following formula was applied to each class:

$$ varPerSNP=\frac{\left(\left({h}^2\div n\right)\times 100\right)}{10^{-4}} $$

Where *h*^*2*^ represents the variance captured by the class (heritability) and is divided by *n,* the total number of variants found in the class. This result was multiplied by 100 to get a percentage of the variances explained by the class and then divided the result by 10^−4^ so that the results can be visually represented.

## Additional files

Additional file 1: Minor Allele Frequencies: Full matrix of the MAF for all classes. (XLSX 13 kb)

Additional file 2: Euclidean matrix with distance values showing the similarities between functional class GRM. The more similar the GRM is between two classes the lower the Euclidean distance measure is. This is also represented by the color green. The more dissimilar the GRM is between two classes the higher the Euclidean distance measure is. Represented by the red color. (XLSX 10 kb)

Additional file 3: The heritability for each trait along with the permutated heritability obtained from the permutation test using the same number but randomly chosen SNPs (which was replicated 5 times and significance is determined as greater or less than the average of the proportion of variance explained by the randomly chosen SNP ± 2 × S.E). The heritability percent difference is simply the difference between the class heritability and the permutated heritability multiplied by 100. (XLSX 13 kb)

Additional file 4: Variance Component Analysis 2: performed using variants with MAF thresholds of 0.000000001 and 0.1. For each functional class is the proportion of variance captured by each class, along with the total proportion of variance captured for each trait represented as a percentage, in brackets. (XLSX 11 kb)

Additional file 5: Variance Component Analysis 2: performed using variants with MAF thresholds of 0.000000001 and 0.1. For each functional class is the variance explained per sequence variant along with the variance component. (XLSX 10 kb)

## Abbreviations

asRNA: Antisense RNA
CDS: Protein coding sequence
CNV: COPY number variants
GRM: Genomic relationship matrix
GWAS: Genome wide association study
Indel: Insertion and deletion
LD: linkage disequilibrium
lncRNA: Long noncoding RNA
MAF: Minor allele frequency
miRNA: microRNA
REML: Restricted maximum likelihood
S.E: Standard error
SNP: Single nucleotide polymorphism
TAV: Trait associated variant
TFBS: Transcription factor binding site
UTR: Untranslated region
wgs: WHOLE genome sequence
